# Early enteral nutrition and dynamic metabolic-inflammatory trajectories in sepsis: a retrospective cohort study

**DOI:** 10.3389/fmed.2026.1817436

**Published:** 2026-04-28

**Authors:** Jing Zhou, Kunping Cui, Quanxiu Tang, Yue Ruan, Xia Li

**Affiliations:** 1 Department of Critical Care Medicine, West China Hospital, Sichuan University, Chengdu, Sichuan, China; 2 West China School of Nursing, Sichuan University, Chengdu, Sichuan, China; 3 Center of Infectious Diseases, West China Hospital of Sichuan University, Chengdu, China

**Keywords:** albumin, biomarker trajectories, critical care, early enteral nutrition, lactate, mortality, procalcitonin, sepsis

## Abstract

**Background:**

Early enteral nutrition (EEN) is an important part of sepsis management, but its physiological effects are not fully understood. This study examined whether EEN influences the time course of metabolic and inflammatory biomarkers in patients with sepsis.

**Methods:**

We performed a retrospective cohort study of 3,354 adult ICU patients with sepsis admitted to West China Hospital from 2011 to 2025. Group-based trajectory modeling was used to characterize longitudinal patterns of albumin, lactate, and procalcitonin. Associations between EEN and trajectory membership were assessed using multinomial logistic regression. The relationship between trajectory groups and 28-day mortality was further evaluated.

**Results:**

Distinct trajectory groups were identified for each biomarker, reflecting heterogeneous nutritional, metabolic, and inflammatory responses. EEN was associated with more favorable albumin trajectories and lower odds of belonging to elevated lactate and procalcitonin patterns. In the multivariate joint trajectory model integrating all three biomarkers, three classes emerged: a stable–low inflammation pattern (67.5%), an intermediate–transient pattern (21.7%), and a high-risk inflammatory surge pattern (10.8%). EEN was independently associated with reduced likelihood of assignment to the intermediate (OR 0.66; 95% CI, 0.52–0.84) and high-risk (OR 0.57; 95% CI, 0.38–0.88) classes. The high-risk trajectory group showed significantly increased 28-day mortality.

**Conclusion:**

Initiation of EEN was linked to a higher probability of remaining in low-risk albumin–lactate–PCT trajectories and a lower probability of entering the high-risk inflammatory surge pattern. This pattern-level shift may partly explain the observed reduction in short-term mortality associated with EEN.

## Introduction

Sepsis remains a major cause of preventable mortality and long-term disability worldwide. The most recent global systematic analysis estimated 166 million sepsis cases and 21.4 million sepsis-related deaths in 2021, representing 31.5% of all global deaths, and showed that the global burden of sepsis increased in 2020 and 2021 after earlier declines. These findings further highlight the need for early supportive strategies in sepsis care (10). Among these, nutrition therapy is considered a cornerstone of sepsis care. Current guidelines, including the Surviving Sepsis Campaign 2021, recommend initiating enteral nutrition within 24–72 h when clinically feasible (9). However, the optimal timing and physiological effects of early enteral nutrition (EEN) remain debated.

Although EEN is widely promoted, clinical evidence remains inconsistent. Several meta-analyses and database studies have reported variable effects on mortality and complications among critically ill and septic patients (5, 11, 36). For example, Elke et al. reported that EEN approaching recommended targets was linked to a significantly lower 60-day mortality (∼20–30% reduced odds) in critically ill sepsis patients (8). Moon et al., in their systematic review, did not identify a significant reduction in mortality with EEN among sepsis patients (22). Some studies have shown that early feeding is associated with shorter ICU stays and lower rates of acute kidney injury, while others have raised concerns about intolerance or adverse events in patients with unstable hemodynamics or mechanical ventilation (13, 35). These inconsistencies may reflect underlying heterogeneity in metabolic and inflammatory responses among septic patients. Understanding how EEN influences the temporal evolution of such physiological processes could help explain these divergent findings (7).

Traditional analyses often rely on single time-point values or simple change scores to assess treatment effects. Yet increasing evidence indicates that sepsis is a dynamic syndrome, and the evolution of host-response biomarkers provides better prognostic information than isolated measurements. For example, studies using group-based or latent-class trajectory models have shown that rapid lactate clearance is closely associated with improved survival, whereas persistently elevated levels indicate poor outcomes (34). Similar findings have been reported for inflammatory biomarkers such as C-reactive protein (CRP) and procalcitonin (PCT), whose temporal trends may predict mortality more accurately than isolated values (15, 33). Albumin trajectories have also been associated with clinical outcomes in sepsis, particularly in distinguishing persistently low from recovering patterns (31). Integrating metabolic and nutritional markers may further enhance prognostic precision. The lactate-to-albumin ratio (LAR), for instance, captures both tissue perfusion and protein synthesis, and persistently high or rising values are related to increased mortality and organ injury in sepsis (26). These results highlight that coupled biomarker dynamics may better reflect the underlying physiological recovery process (12). A recent review also indicates that the detection and diagnosis of sepsis need the combined application of multiple biomarkers (4).

However, most studies of EEN focus on clinical endpoints without examining how nutrition influences the time course of metabolic and inflammatory responses. Few have analyzed whether EEN is associated with the joint trajectories of biomarkers such as lactate, albumin, and PCT (36). Addressing this gap is one of the main novel aspects of the present study. Furthermore, trajectory-based endpoints have rarely been explored as potential mediators linking nutrition to mortality. Evidence from Asian critical care settings is also limited, and findings derived from Western critical care databases such as the Medical Information Mart for Intensive Care IV (MIMIC-IV), including trajectory-based sepsis studies, may not be fully generalizable to different healthcare systems and patient populations (16, 20, 21).

To address these gaps, we conducted a longitudinal analysis of adult ICU patients with sepsis using routinely collected electronic health record data from the West China Hospital Big Data Platform. We characterized the trajectories of albumin, lactate, and procalcitonin, and then applied a multivariate trajectory model to identify combined metabolic–inflammatory patterns. We further examined whether EEN within 48 h of ICU admission was associated with more favorable trajectories and whether these trajectory groups were linked to short-term mortality.

## Materials and methods

### Design, setting, and participants

This retrospective cohort study utilized electronic health record data sourced from the West China Hospital Big Data Platform of Sichuan University, a centralized repository that integrates clinical information from West China Hospital and affiliated medical consortiums across multiple provinces in western China (32). As the national center for diagnosis and treatment of complex critical illnesses in western China, West China Hospital serves as a tertiary referral hub, drawing challenging cases from surrounding regions and enriching the dataset with diverse, high-volume patient encounters (19). We queried the platform for adult patients diagnosed with sepsis between January 2011 and July 2025, yielding a cohort to evaluate the impact of EEN on joint trajectories of albumin, lactate, and related outcomes. Patients were included if they met the following criteria: (1) a clinical diagnosis of sepsis recorded in the electronic health record, based on routine clinical assessment using Sepsis-3 criteria; (2) age ≥18 years; (3) hospital length of stay between 48 h and 30 days; and (4) at least three repeated measurements of serum albumin, lactate, and procalcitonin during hospitalization. Because this was a retrospective study based on routine clinical care, biomarker measurements were obtained according to clinical judgment rather than a prespecified study schedule. The timing of all longitudinal measurements was indexed to time since hospital admission. For trajectory analyses, only biomarker measurements obtained within the first 7 days after hospital admission were included, in order to characterize early treatment-phase dynamics rather than later convalescent-phase changes. Exclusion criteria comprised age <18 years, hospital stay <48 h or >30 days, or fewer than three measurements for any of the aforementioned biomarkers. This study was approved by the Ethics Committee of West China Hospital, Sichuan University, with a waiver of informed consent (No. 2025–907).

## Variables

### Exposure, outcome and covariates

The exposure of interest was EEN, defined as initiation of enteral feeding within 48 h of ICU admission, rather than from the time of sepsis diagnosis, because ICU admission time was more consistently recorded and provided a more stable temporal anchor in this retrospective EHR-based study. The control group was defined as patients who did not receive enteral nutrition within this early window, including both those who initiated enteral feeding more than 48 h after ICU admission and those who did not receive enteral nutrition during the observation period. Patients were therefore classified into an EEN group and a non-EEN group according to whether enteral nutrition was initiated within this 48-h window. The 48-h definition aligns with recent critical-care nutrition studies and systematic reviews focused on sepsis or septic shock, which evaluate within 48 h as a pragmatic early window (11).

The primary outcome was 28-day in-hospital mortality, defined as death occurring within 28 days after hospital admission during the index hospitalization and ascertained from hospital discharge and death records. The secondary outcome was all-cause in-hospital mortality during the index admission. Adjustment variables were prespecified based on clinical plausibility and prior evidence for confounding in nutrition–outcome associations in sepsis, and were measured at baseline on hospital day 1 before or at enteral nutrition initiation. Covariates included age, sex, ethnicity, education, hypertension, diabetes, coronary heart disease, chronic kidney disease, and first-day laboratory values for albumin, lactate, C-reactive protein, and PCT.

### Health trajectory indicators

Longitudinal trajectories were modeled for serum albumin as an index of nutritional status and hepatic synthetic function, serum lactate as a marker of global hypoperfusion and metabolic stress, and PCT as a marker of infection-driven inflammatory activity (31, 33, 34). These indicators were selected because dynamic patterns add prognostic information beyond static values in sepsis cohorts, with multiple studies showing that lactate trajectories stratify short-term mortality risk, that PCT trajectories identify high-risk phenotypes, and that coupling metabolic with nutritional signals improves discrimination, including evidence on the lactate-to-albumin axis and its kinetics. Because this study used routinely collected clinical data, biomarker measurements were irregular and not fully synchronized across indicators or patients. To enable joint longitudinal analysis, all biomarker observations were aligned using the common time scale of time since hospital admission. Only measurements recorded within the first 7 days after hospital admission were included in the trajectory analyses. To reduce skewness, lactate was analyzed on the log scale and PCT as log (value + 1).

### Statistical analysis

Baseline characteristics were summarized according to EEN exposure. Continuous variables were reported as medians with interquartile ranges, and categorical variables as counts and percentages. Continuous variables were compared using Student’s t test or the Mann–Whitney U test, as appropriate, and categorical variables were compared using the χ^2^ test or Fisher’s exact test, as appropriate. To address confounding by indication, we estimated the probability of receiving EEN using logistic regression including all covariates specified above. Missing values were imputed using medians for continuous variables and modal categories for categorical variables. Patients were matched 1:1 using nearest-neighbor matching without replacement and a caliper of 0.2 on the logit of the propensity score. Balance between groups was assessed using standardized mean differences (SMDs), with SMD < 0.10 indicating adequate balance. All analyses of EEN effects were performed in the matched cohort (1).

To explore longitudinal patterns of key biomarkers, group-based trajectory modeling was performed separately for lactate, albumin, and PCT. For each marker we fitted models including linear, quadratic, and cubic time terms and allowed a maximum of five trajectory classes. The optimal number of trajectory groups was determined by three criteria: (1) mean posterior probability of class membership of at least 0.70 for every class, (2) each class representing at least 2% of the total study population, and (3) the lowest Bayesian Information Criterion (BIC) indicating best overall model fit (18, 23). After single-marker modeling, we applied a group-based multivariate trajectory (GBMT) approach to construct joint trajectories that captured the combined evolution of lactate, albumin, and PCT. In the joint trajectory analysis, competing 3–5 class solutions were evaluated based on the single-biomarker trajectory results and clinical interpretability. For trajectory modeling, all biomarker values were analyzed on the shared time scale of time since hospital admission within the first 7 days after admission. The association between EEN and trajectory membership was assessed using multinomial logistic regression to account for multiple trajectory categories simultaneously. Three sequential models were constructed to examine the robustness of the association under progressive adjustment for potential confounders. Model 1 included EEN as the sole independent variable, representing the crude association. Model 2 was adjusted for demographic characteristics, including age and sex. Model 3, the fully adjusted model, additionally incorporated major comorbidities known to influence nutritional tolerance and sepsis outcomes, including hypertension, diabetes mellitus, coronary heart disease, and chronic kidney disease. Covariates in Model 3 were prespecified on the basis of clinical relevance and prior literature. To explore the clinical implications of trajectory heterogeneity, logistic regression analyses were further conducted to estimate odds ratios (ORs) and 95% confidence intervals (CIs) for in-hospital mortality across the identified trajectory groups, using the most favorable trajectory as the reference. Among these, Model 3 served as the primary adjustment framework for evaluating the association between trajectory patterns and mortality, providing the most conservative and clinically interpretable estimates.

All statistical analyses were performed using R software (version 4.2.0; R Foundation for Statistical Computing, Vienna, Austria) and Stata/MP (version 18.0; StataCorp LLC, College Station, TX, United States). The single-biomarker trajectory models were estimated using the lcmm package in R, whereas the joint group-based multivariate trajectory model was implemented using the traj procedure in Stata. These software packages were used for different trajectory analyses rather than for cross-validation of the same model. A two-sided *p* value of less than 0.05 was considered statistically significant.

## Results

**Figure 1:**
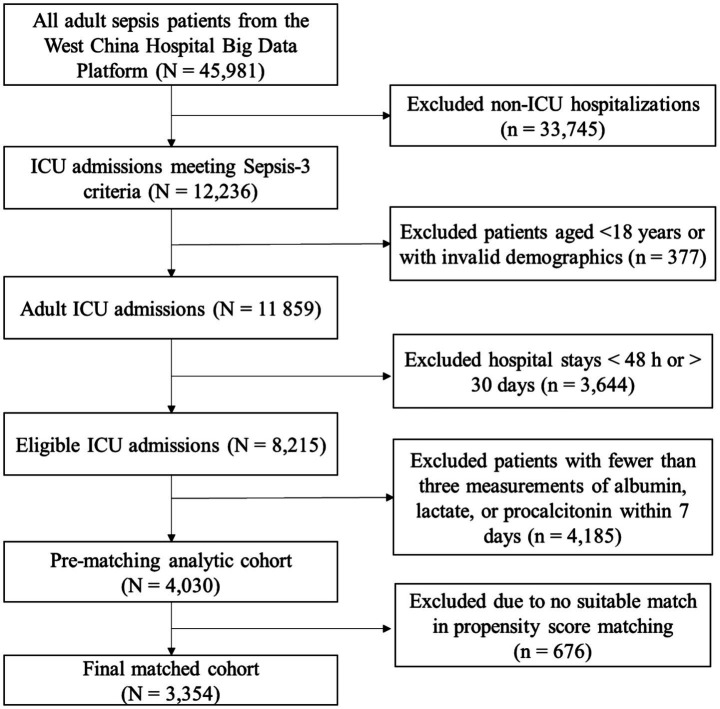
Flow diagram of the study.

The study initially identified 45,981 patients with sepsis from the West China Hospital Big Data Platform. After excluding non-ICU hospitalizations, pediatric cases, short or prolonged stays, and patients without sufficient biomarker measurements, 4,030 patients remained eligible before propensity score matching. After 1:1 propensity score matching, 3,354 patients (1,677 per group) were included in the final analysis (Figure 1).

Table 1 summarizes the baseline characteristics of the matched cohort, with 1,677 patients in each group. The distributions of sex, marital status, educational attainment, ethnicity, and major comorbidities, including hypertension, diabetes mellitus, coronary heart disease, and chronic kidney disease, were generally similar between groups. Initial laboratory values, including serum albumin, C-reactive protein, and procalcitonin, were also broadly comparable. However, small residual differences remained for several variables, including age, BMI, and baseline lactate.

**Table 1:** Baseline characteristics after propensity score matching, stratified by EEN.

Characteristic	Level	No EEN (*n* = 1,677)	EEN (*n* = 1,677)	*p*-value
Sex, *n* (%)	Male	1,174 (70.0)	1,185 (70.7)	0.705
	Female	503 (30.0)	492 (29.3)	
Age (mean ± SD)		61.38 (16.31)	62.52 (16.66)	0.045
BMI, median [IQR]		22.60 [20.89, 24.77]	22.49 [20.76, 24.49]	0.034
Marital status, *n* (%)	Divorced/Widowed	108 (6.4)	128 (7.6)	0.706
	Other	7 (0.4)	5 (0.3)	
	Single	82 (4.9)	79 (4.7)	
	Married	1,479 (88.2)	1,464 (87.3)	
Education, *n* (%)	Primary	363 (21.6)	320 (19.1)	0.093
	Higher	349 (20.8)	390 (23.3)	
	Other	244 (14.5)	222 (13.2)	
	Secondary	721 (43.0)	745 (44.4)	
Ethnicity, *n* (%)	Tibetan	67 (4.0)	57 (3.4)	0.259
	Han	1,418 (84.6)	1,453 (86.6)	
	Other minorities	175 (10.4)	157 (9.4)	
	Yi	17 (1.0)	10 (0.6)	
Hypertension, *n* (%)	No	1,081 (64.5)	1,035 (61.7)	0.107
	Yes	596 (35.5)	642 (38.3)	
Diabetes, *n* (%)	No	1,157 (69.0)	1,141 (68.0)	0.577
	Yes	520 (31.0)	536 (32.0)	
Coronary heart disease, *n* (%)	No	1,622 (96.7)	1,620 (96.6)	0.923
	Yes	55 (3.3)	57 (3.4)	
Chronic kidney disease, *n* (%)	No	1,580 (94.2)	1,571 (93.7)	0.562
	Yes	97 (5.8)	106 (6.3)	
Albumin (mean ± SD)		30.60 (6.38)	30.56 (5.33)	0.87
Lactate, median [IQR]		1.70 [1.30, 2.40]	1.70 [1.30, 2.40]	0.014
C-reactive protein, median [IQR]		113.00 [52.40, 186.00]	103.00 [55.70, 169.00]	0.054
Procalcitonin, median [IQR]		1.54 [0.41, 6.03]	1.34 [0.37, 5.51]	0.14
WBC count, median [IQR]		10.05 [6.57, 14.70]	10.66 [7.20, 14.82]	0.016

Values are expressed as mean ± SD, median [IQR], or *n* (%), as appropriate. Continuous variables were compared using Student’s *t*-test or Mann–Whitney U test; categorical variables were compared using *χ*^2^ test or Fisher’s exact test, as appropriate. ^a^Other minorities include Hui, Manchu, Qiang, Tujia, Bai, and other ethnic groups.

### Results of single-biomarker trajectory analysis

**Figure 2:**
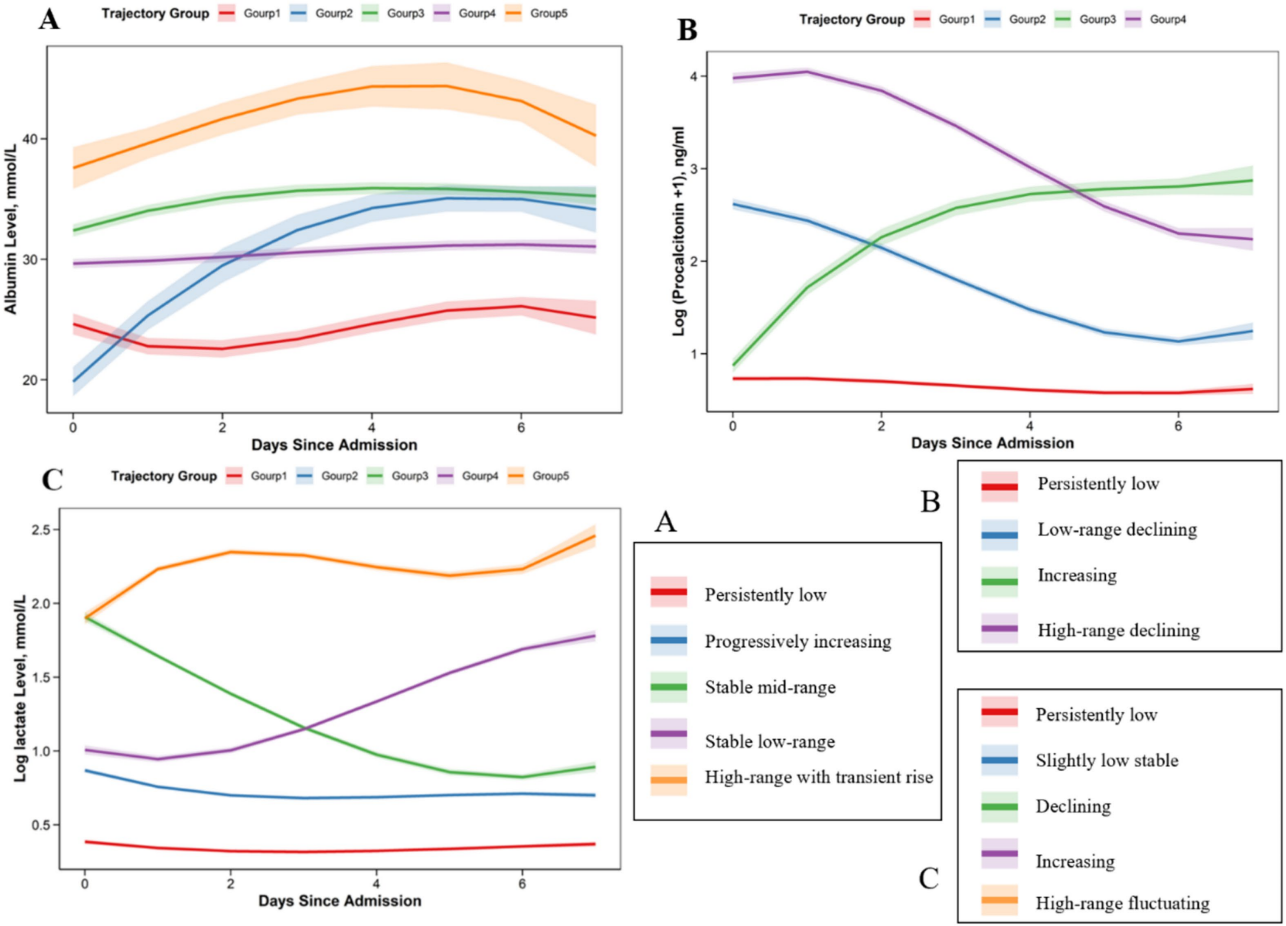
Single-biomarker trajectory groups for albumin, procalcitonin, and lactate in patients with sepsis. **(A–C)** Show the estimated trajectory curves for albumin, procalcitonin, and log-lactate, respectively, over the first 7 days after hospital admission. Each colored line represents one trajectory group identified by group-based trajectory modeling.

For serum albumin, five trajectory groups were identified, differing mainly in baseline level and change over time: a persistently low pattern, a progressively increasing pattern, a stable mid-range pattern, a stable low-range pattern, and a high-range pattern with a modest transient rise. For log-lactate, five distinct trajectories were observed: a persistently low pattern, a slightly low stable pattern, a declining pattern, an increasing pattern, and a high-range fluctuating pattern. For log (PCT + 1), four trajectory groups were identified: a persistently low pattern, a low-range declining pattern, an increasing pattern, and a high-range declining pattern. Overall, these trajectory groups differed in both baseline level and direction of change over time (Figure 2). The selected solutions showed the best overall balance of model fit, classification quality, and minimum class size among the competing models (Supplementary Tables 1–3).

### Association of EEN with single-biomarker trajectories

**Figure 3:**
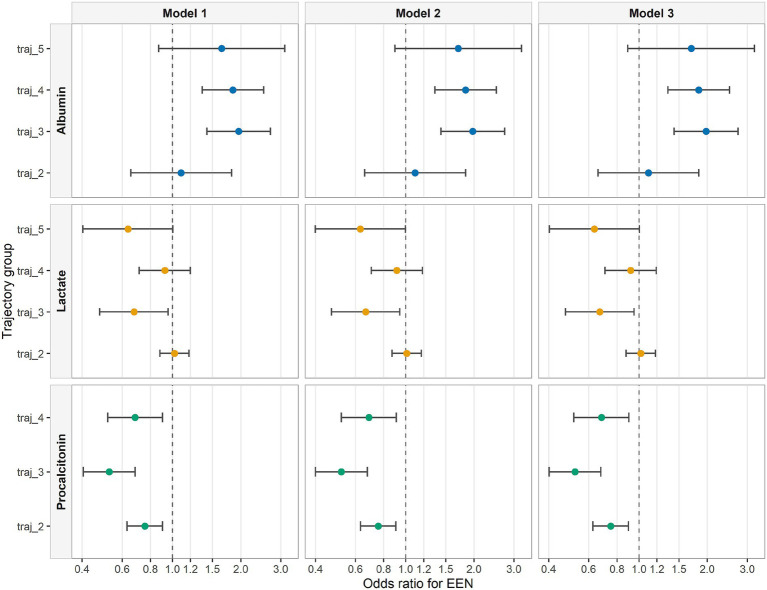
Association between EEN and single-biomarker trajectory groups. Odds ratios and 95% confidence intervals were estimated using multinomial logistic regression, with trajectory 1 as the reference group. Model 1 was unadjusted, model 2 was adjusted for age and sex, and model 3 was additionally adjusted for hypertension, diabetes mellitus, coronary heart disease, and chronic kidney disease.

Multinomial logistic regression analyses were performed to evaluate the association between EEN and trajectory membership. The results are depicted in Figure 3. Regarding albumin, using the lowest baseline group (Trajectory 1, persistently low albumin) as the reference, EEN was significantly associated with favorable trajectory patterns. In the fully adjusted model, patients receiving EEN had nearly double the odds of belonging to Trajectory 3 (stable mid-range albumin; OR 1.98, 95% CI 1.43–2.73, *p* < 0.001) and Trajectory 4 (stable low-range albumin; OR 1.84, 95% CI 1.34–2.51, p < 0.001). No significant association was observed for Trajectory 2 (progressively increasing albumin) or Trajectory 5 (high-range albumin with transient rise). For lactate, using Trajectory 1 (persistently low lactate) as the reference, EEN was associated with lower odds of belonging to Trajectory 3 (declining lactate; OR 0.67, 95% CI 0.47–0.95, *p* = 0.025). A similar pattern was observed for Trajectory 5 (high-range fluctuating lactate; OR 0.64, 95% CI 0.40–1.00, *p* = 0.052), although this did not reach statistical significance. For procalcitonin, using Trajectory 1 (persistently low PCT) as the reference, EEN was associated with lower odds of belonging to Trajectory 2 (low-range declining PCT; OR 0.75, 95% CI 0.63–0.90, *p* = 0.002), Trajectory 3 (increasing PCT; OR 0.52, 95% CI 0.40–0.68, *p* < 0.001), and Trajectory 4 (high-range declining PCT; OR 0.68, 95% CI 0.52–0.90, *p* = 0.007). All results in detail can be found the Supplementary Table 4.

### Results of joint trajectory analysis of multiple biomarkers

**Figure 4:**
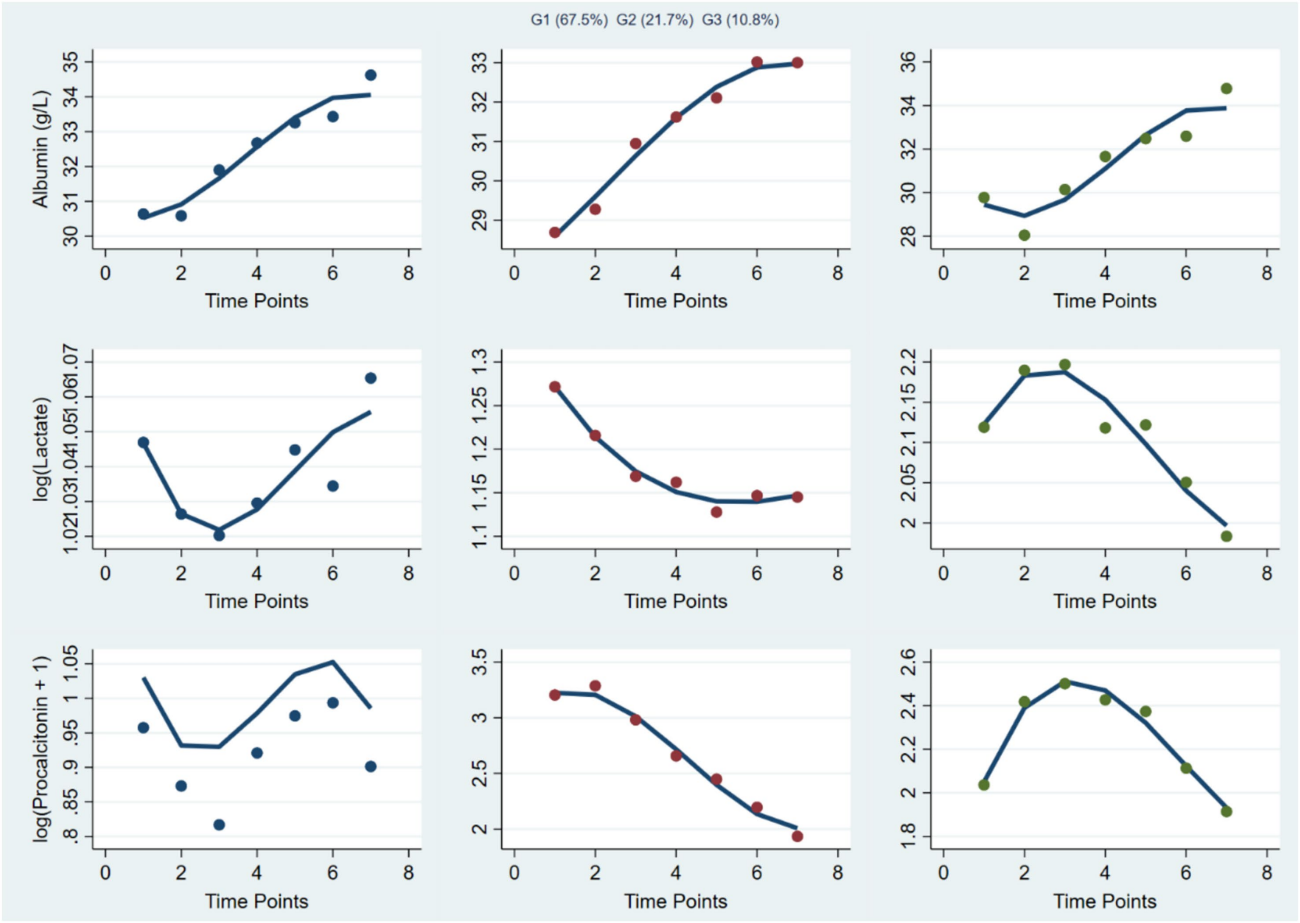
Joint trajectory classes integrating albumin, lactate, and procalcitonin in patients with sepsis. The figure shows the estimated joint trajectories of the three indicators over the first 7 days after hospital admission. Three latent classes were identified: Stable–low inflammation (SLI), intermediate–transient inflammation (ITI), and high-risk–inflammatory surge (HR-IS).

We identified a three-class group-based multi-trajectory model for albumin, lactate, and PCT on the basis of superior BIC and high classification quality (entropy ≈ 0.94). The latent classes were labeled Stable–Low Inflammation (SLI), Intermediate–Transient Inflammation (ITI), and High-Risk–Inflammatory Surge (HR-IS). The SLI class (~67.5%) featured higher, stable albumin with mild curvature and low, declining log-lactate and log-PCT, indicating persistently low inflammatory activity. The ITI class (~21.7%) showed mid-range albumin, a gentle decrease in log-lactate, and a shallow hump-shaped pattern in log-PCT (early rise followed by normalization), consistent with a transient inflammatory response. The HR-IS class (~10.8%) was characterized by lower albumin with an initial dip and partial recovery alongside elevated, early-rising log-lactate and log-PCT that later decelerated or plateaued, reflecting an acute inflammatory surge. Average posterior probabilities were high across classes, supporting reliable class assignment (Figure 4). All the detail results can be found in Supplementary Tables 5–7.

### Association between EEN and joint biomarker trajectories

**Figure 5:**
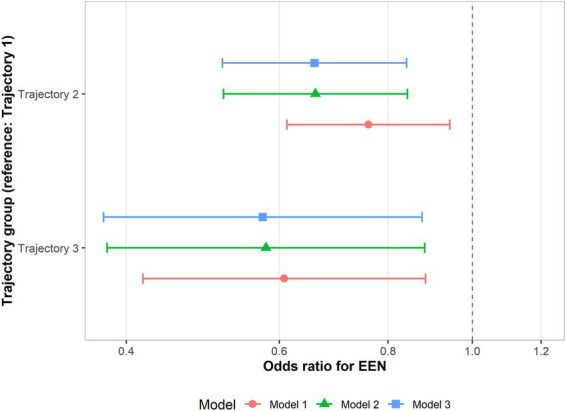
Association between EEN and joint trajectory groups. Odds ratios and 95% confidence intervals were estimated using multinomial logistic regression, with the Stable–Low Inflammation (SLI) group as the reference. Model 1 was unadjusted, model 2 was adjusted for age and sex, and model 3 was additionally adjusted for hypertension, diabetes mellitus, coronary heart disease, and chronic kidney disease.

Multivariable multinomial logistic regression was employed to determine the independent association between EEN and joint trajectory membership, using the Stable–Low Inflammation group (Trajectory 1) as the reference. In the fully adjusted analysis (Model 3), EEN was associated with a 34% reduction in the odds of membership in the Intermediate–Transient Inflammation group (Trajectory 2: OR 0.66, 95% CI 0.52–0.84, *p* < 0.001). Furthermore, EEN was associated with lower odds of membership in the High-Risk–Inflammatory Surge phenotype (Trajectory 3), reducing the odds of membership by approximately 43% (OR 0.57, 95% CI 0.38–0.88, *p* = 0.010). These findings suggest that early enteral feeding was associated with a lower likelihood of belonging to more adverse metabolic–inflammatory trajectory groups (Figure 5). All the detail results are displayed in Supplementary Table 8.

### Association between trajectory patterns and mortality outcomes

**Figure 6:**
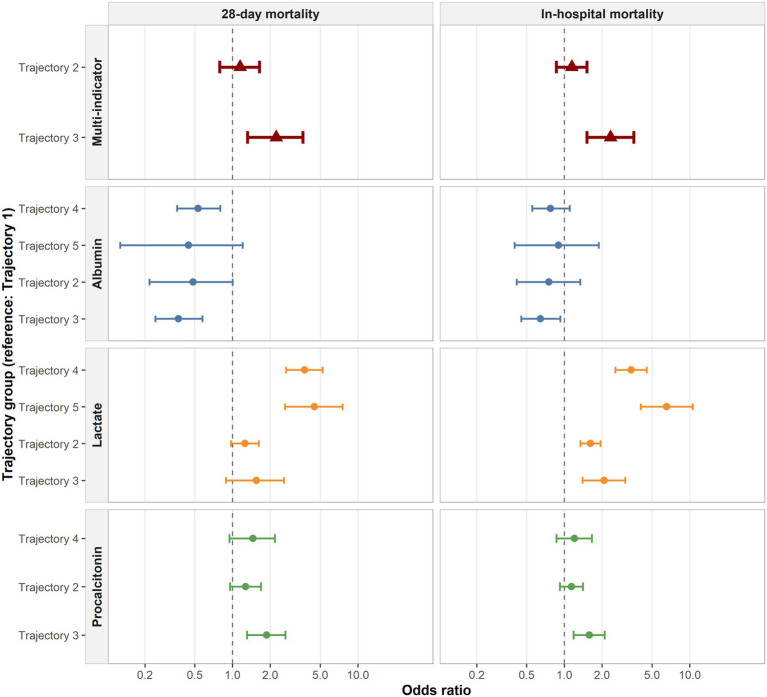
Association between trajectory patterns and death outcome. OR and 95% confidence intervals were estimated using logistic regression. For each analysis, trajectory 1 was used as the reference.

Logistic regression analyses evaluated the association between biomarker trajectories and mortality outcomes (Figure 6). For albumin, compared with the reference group (Trajectory 1), Trajectory 3 and Trajectory 4 were associated with significantly lower odds of 28-day mortality (OR 0.37, 95% CI 0.24–0.58, *p* < 0.001; and OR 0.53, 95% CI 0.36–0.80, *p* = 0.002, respectively). No significant associations were observed for Trajectory 2 or Trajectory 5 regarding 28-day mortality. For lactate, Trajectory 4 and Trajectory 5 were significantly associated with increased 28-day mortality, with ORs of 3.76 (95% CI 2.68–5.25, *p* < 0.001) and 4.51 (95% CI 2.63–7.54, *p* < 0.001), respectively. While Trajectories 2 and 3 were not statistically significant for 28-day mortality, all lactate trajectory groups (Trajectories 2–5) were significantly associated with increased in-hospital all-cause mortality compared with the reference. For procalcitonin, only Trajectory 3 showed a significant association with 28-day mortality (OR 1.88, 95% CI 1.31–2.65, *p* < 0.001) and all-cause mortality (OR 1.59, 95% CI 1.19–2.10, *p* = 0.002). Trajectories 2 and 4 did not differ significantly from the reference group.

In the joint multi-biomarker analysis, Trajectory 3 (High-Risk–Inflammatory Surge) was significantly associated with higher 28-day mortality (OR 2.23, 95% CI 1.32–3.64, *p* = 0.002) and all-cause mortality (OR 2.34, 95% CI 1.51–3.58, *p* < 0.001). Trajectory 2 (Intermediate–Transient Inflammation) showed no significant difference in mortality risk compared with the reference group (*p* > 0.34; Supplementary Table 9).

## Discussion

In this large real-world cohort, EEN was associated with lower-risk metabolic–inflammatory trajectories across albumin, lactate, and procalcitonin. Importantly, the joint trajectory analysis demonstrated that EEN was linked to a reduced probability of belonging to the high-risk inflammatory surge phenotype, which was strongly associated with short-term mortality. These findings suggest that the effects of nutritional timing may be better understood through the dynamic evolution of host-response patterns rather than isolated biomarker values.

Prior studies evaluating EEN have reported heterogeneous outcomes, with some analyses suggesting improved survival or organ function and others showing limited benefit or uncertainty regarding patient tolerance (2, 11, 35, 36). The Surviving Sepsis Campaign 2021 recommends initiating enteral nutrition within 72 h in hemodynamically stable patients (9), whereas more recent critical-care nutrition studies support an even earlier window, within 24–48 h, as both feasible and safe when shock control is achieved (13, 14). Our data, demonstrating a protective association for EEN within 48 h, are consistent with this emerging evidence. This trajectory-based perspective may help reconcile prior conflicting findings by suggesting that EEN does not uniformly reduce mortality across all patients, but instead confers benefit primarily by shifting individuals away from high-inflammatory, high-risk biomarker trajectories and attenuating the persistence of metabolic and inflammatory disturbances over time.

From a biomarker perspective, our trajectory-based findings align with recent research emphasizing the prognostic value of dynamic trends over single measurements. Wei et al. demonstrated that distinct lactate trajectories predict 28-day mortality independent of baseline values (34). Wang et al. reported that persistent high PCT trajectories identify patients at greatest risk (33). Likewise, Tie et al. showed that recovery patterns of albumin are more prognostic than single measures (31). Building on these findings, our study extends the analysis from individual biomarkers to joint trajectory modeling encompassing metabolic, inflammatory, and nutritional domains. This multivariate framework captures the covariance and interdependence among host responses, providing a more integrated view of sepsis pathophysiology. It also accounts for additional variability in mortality outcomes that cannot be explained by single-biomarker analyses alone.

The three latent trajectory classes identified in this study are conceptually analogous to the adaptive, intermediate, and hyperinflammatory subphenotypes described in previous latent-class analyses of sepsis, which revealed reproducible patterns of immune activation and clinical outcomes (3, 6, 29). Patients in our High-Risk–Inflammatory Surge group exhibited biochemical features resembling hyperinflammatory phenotypes, including sustained inflammation, metabolic stress, and catabolic signatures characterized by low albumin and high lactate and PCT levels. Importantly, patients receiving EEN were less likely to follow this adverse trajectory, suggesting a potential biological interaction between nutritional timing and host-response evolution. Although direct causal evidence is still limited, this observation is biologically plausible and aligns with prior mechanistic studies showing that timely enteral feeding can stabilize intestinal integrity, modulate the gut–liver–immune axis, and temper excessive systemic inflammation (9, 13, 14, 33).

Several biological mechanisms may underlie the association between EEN and favorable metabolic–inflammatory trajectories. First, serum albumin in sepsis should be interpreted cautiously, as it is influenced not only by nutritional support and hepatic protein synthesis, but also by systemic inflammation, vascular permeability, and fluid balance. Accordingly, the more favorable albumin trajectories observed in the EEN group may reflect a combination of nutritional support and broader clinical recovery, rather than a nutrition-specific effect alone (27, 30). Second, adequate early feeding may help preserve splanchnic blood flow and oxygen utilization following hemodynamic stabilization, thereby facilitating lactate clearance and preventing metabolic acidosis (25). Third, EEN exerts immunometabolism modulation through the gut–liver axis, maintaining epithelial barrier integrity, reducing bacterial translocation, and tempering systemic inflammatory signaling (24, 28, 30). These effects correspond with the observed downward trends in inflammatory markers among patients receiving early feeding. Furthermore, composite indicators integrating metabolic and nutritional balance, such as the lactate-to-albumin ratio (LAR), have been repeatedly associated with sepsis outcomes (17, 37). Collectively, these mechanisms are consistent with the possibility that EEN may be associated with a shift toward a lower-risk metabolic–inflammatory profile.

From a clinical standpoint, trajectory-based assessment offers several advantages. First, it provides a dynamic indicator of treatment response: patients remaining in high-risk lactate or multi-indicator trajectories may benefit from early reassessment of hemodynamics, source control, or nutritional adequacy. Second, identifying trajectory groups that are responsive to EEN may help refine patient selection, enabling more targeted nutritional strategies in the early phase of sepsis. Third, integrating biomarker trajectories into routine monitoring could support real-time risk stratification, complementing established tools such as SOFA or lactate clearance (28).

This study has several strengths. First, it draws on a large longitudinal cohort with detailed serial biomarker measurements, which allowed us to construct well-separated trajectory classes. Second, by jointly modeling albumin, lactate, and PCT, we captured nutritional, perfusion-related, and infection-related processes within a single framework, providing a more integrated view of sepsis biology than most prior EEN studies. Third, we combined single-indicator and multi-indicator models, and used propensity-score matching to reduce baseline imbalances between patients who did and did not receive early feeding.

Several limitations should be acknowledged. First, despite propensity-score matching and multivariable adjustment, residual confounding cannot be excluded in this retrospective observational study; moreover, infectious focus was not uniformly available in structured form, and structured SOFA and APACHE data were too incomplete to be included as baseline severity covariates. In addition, because cohort identification relied on sepsis diagnoses recorded in routine clinical care rather than *de novo* adjudication using complete SOFA data, some misclassification of sepsis status cannot be entirely excluded. Second, the non-EEN group combined patients with delayed enteral nutrition and those without enteral nutrition, which may have introduced clinical heterogeneity, and some residual collinearity among adjusted comorbidities cannot be ruled out. Third, biomarker measurements were obtained from routine clinical care rather than a prespecified protocol, resulting in variation in measurement timing and frequency. Fourth, we examined the timing of EEN initiation but did not evaluate nutritional adequacy, such as calorie and protein delivery, which should be incorporated as time-varying exposures in future studies. Finally, trajectory modeling identifies dynamic biological patterns but does not establish causality; therefore, these findings require confirmation in prospective and interventional studies.

## Conclusion

In summary, our findings highlight the importance of longitudinal biomarker dynamics in understanding the potential benefits of EEN. EEN was associated with a shift toward more favorable metabolic–inflammatory trajectories, and high-risk trajectory groups were strongly predictive of short-term and in-hospital mortality. These results support incorporating trajectory-based assessment into sepsis monitoring and raise the possibility that nutritional support may influence clinical outcomes through its effects on physiologic evolution. Prospective studies are needed to validate these observations and to determine whether tailoring EEN to trajectory phenotypes can improve survival.

## Data Availability

The data analyzed in this study is subject to the following licenses/restrictions: The de-identified data supporting the findings of this study are available from the corresponding author upon reasonable request and with institutional approval. Requests to access these datasets should be directed to Xia Li, lixia4040@163.com.
